# Machine learning algorithm to predict anterior cruciate ligament revision demonstrates external validity

**DOI:** 10.1007/s00167-021-06828-w

**Published:** 2022-01-01

**Authors:** R. Kyle Martin, Solvejg Wastvedt, Ayoosh Pareek, Andreas Persson, Håvard Visnes, Anne Marie Fenstad, Gilbert Moatshe, Julian Wolfson, Martin Lind, Lars Engebretsen

**Affiliations:** 1 Department of Orthopedic Surgery, University of Minnesota, 2512 South 7th Street, Suite R200, Minneapolis, MN 55455 USA; 2 Department of Orthopaedic Surgery, CentraCare, Saint Cloud, MN USA; 3 Division of Biostatistics, School of Public Health, University of Minnesota, Minneapolis, MN USA; 4 Department of Orthopedic Surgery, Mayo Clinic, Rochester, MN USA; 5 Norwegian Knee Ligament Register, Haukeland University Hospital, Bergen, Norway; 6 Oslo Sport Trauma Research Center, Norwegian School of Sports Science, Oslo, Norway; 7 Orthopaedic Clinic, Oslo University Hospital Ullevål, Oslo, Norway; 8 Aarhus University Hospital, Aarhus, Denmark

**Keywords:** Machine learning, Artificial intelligence, ACL Reconstruction, ACL revision, Outcome prediction

## Abstract

**Purpose:**

External validation of machine learning predictive models is achieved through evaluation of model performance on different groups of patients than were used for algorithm development. This important step is uncommonly performed, inhibiting clinical translation of newly developed models. Machine learning analysis of the Norwegian Knee Ligament Register (NKLR) recently led to the development of a tool capable of estimating the risk of anterior cruciate ligament (ACL) revision (https://swastvedt.shinyapps.io/calculator_rev/). The purpose of this study was to determine the external validity of the NKLR model by assessing algorithm performance when applied to patients from the Danish Knee Ligament Registry (DKLR).

**Methods:**

The primary outcome measure of the NKLR model was probability of revision ACL reconstruction within 1, 2, and/or 5 years. For external validation, all DKLR patients with complete data for the five variables required for NKLR prediction were included. The five variables included graft choice, femur fixation device, KOOS QOL score at surgery, years from injury to surgery, and age at surgery. Predicted revision probabilities were calculated for all DKLR patients. The model performance was assessed using the same metrics as the NKLR study: concordance and calibration.

**Results:**

In total, 10,922 DKLR patients were included for analysis. Average follow-up time or time-to-revision was 8.4 (± 4.3) years and overall revision rate was 6.9%. Surgical technique trends (i.e., graft choice and fixation devices) and injury characteristics (i.e., concomitant meniscus and cartilage pathology) were dissimilar between registries. The model produced similar concordance when applied to the DKLR population compared to the original NKLR test data (DKLR: 0.68; NKLR: 0.68–0.69). Calibration was poorer for the DKLR population at one and five years post primary surgery but similar to the NKLR at two years.

**Conclusion:**

The NKLR machine learning algorithm demonstrated similar performance when applied to patients from the DKLR, suggesting that it is valid for application outside of the initial patient population. This represents the first machine learning model for predicting revision ACL reconstruction that has been externally validated. Clinicians can use this in-clinic calculator to estimate revision risk at a patient specific level when discussing outcome expectations pre-operatively. While encouraging, it should be noted that the performance of the model on patients undergoing ACL reconstruction outside of Scandinavia remains unknown.

**Level of evidence:**

III.

**Supplementary Information:**

The online version contains supplementary material available at 10.1007/s00167-021-06828-w.

## Introduction

At the time of primary surgery, how does a surgeon estimate the risk of their patient needing a revision anterior cruciate ligament (ACL) reconstruction in the future? Numerous studies have defined failure rate epidemiology and identified risk factors such as age [13, 18, 24, 27, 32, 33], graft choice [13, 18, 21] and size [1], activity level [13, 33], body composition [27], ligamentous laxity [14, 18], and tibial slope [10, 31]. Despite this mass of knowledge, the ability to synthesize it and accurately quantify revision risk at a patient-specific level remains elusive and is often influenced by surgeon experience. This uncertainty is rooted in the complex relationships between the known (and unknown) risk factors that may be present to varying degrees in the patient seated in the office. The personal experience of the surgeon combined with their subjective interpretation of these variables in real time leads to the equivalent of an educated guess regarding revision rate.

Machine learning has the potential to add clarity and improve our predictive capability. While relatively new to knee ligament surgery, the application of machine learning is rapidly transforming clinical care in several fields, including orthopaedic surgery. In short, machine learning is a combination of advanced statistical techniques that can interpret large data sets that are more complex than would be possible with traditional statistics. Through analysis of large databases, machine learning can decipher the complex interactions between variables and generate algorithms capable of outcome prediction. Often, the result is accuracy that is comparable to or better than the prediction of experts in the field [5, 8, 23, 25, 26, 29, 34].

**Fig. 1:**
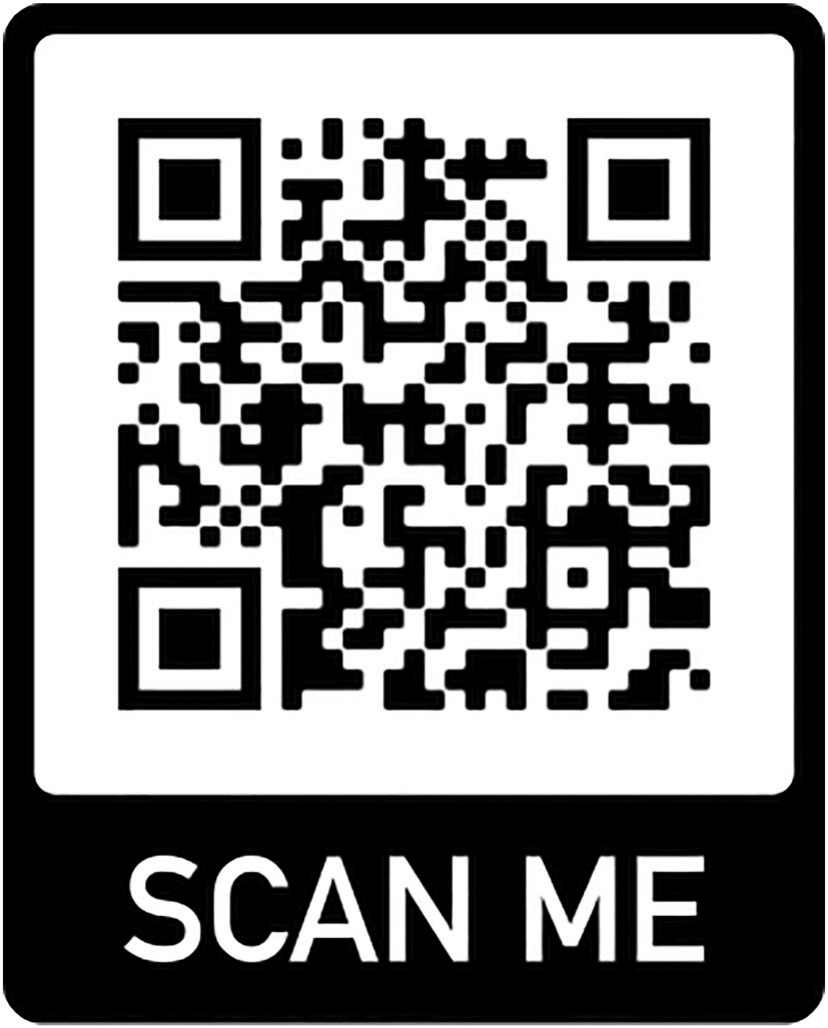
Link to ACL revision risk prediction in-clinic calculator [19]

Recently, machine learning was used to develop a tool that can quantify revision risk for a patient undergoing primary ACL reconstruction (https://swastvedt.shinyapps.io/calculator_rev/; Fig. 1)[19]. The source of data included nearly 25,000 patients with primary ACL reconstruction recorded in the Norwegian Knee Ligament Register (NKLR). The result was a well-calibrated tool capable of predicting revision risk one, two, and five years after primary ACL reconstruction with moderate accuracy. Following model development, external validation is the next step toward clinical application of new models.

The purpose of this study was to determine the external validity of the previously published NKLR ACL revision algorithm by assessing its performance when applied to patients from the Danish Knee Ligament Registry (DKLR). The hypothesis was that model performance would be similar, suggesting validity of the algorithm. This represents the first study to assess external validation of a clinical tool developed using machine learning techniques for outcome prediction following ACL reconstruction. The ability to estimate revision risk at a patient specific level may help guide discussion surrounding outcome expectations pre-operatively.

## Materials and methods

This manuscript was written in accordance with the Transparent Reporting of a multivariable prediction model for Individual Prognosis Or Diagnosis (TRIPOD) statement [6]. The TRIPOD statement is a comprehensive set of recommendations for studies that develop and/or validate prediction models. The 22-item checklist aims to improve transparency of prediction model studies through full and clear information reporting, independent of study methods.

### Ethics

At the time of enrollment in the NKLR all patients provide informed consent and the Norwegian Data Inspectorate grants permission for the register to collect, analyze, and publish on health data. Data registration was performed confidentially according to Norwegian and European Union (EU) data protection rules, with all data de-identified prior to retrieval for analysis. The Regional Ethics Committee (REK) states that it is not necessary to obtain further ethical approval for Norwegian register-based studies [9]. Similarly, the DKLR obtains informed consent at the time of enrollment and patient data was de-identified prior to retrieval for analysis with no further ethical approval required.

### Data source

Original prediction model development was based on machine learning analysis of patients contained within the NKLR while model validation was performed using patients from the DKLR. Both national knee ligament registries prospectively enrol patients undergoing cruciate ligament reconstruction pre-operatively and record demographic, injury, surgical, and follow-up outcome details including subsequent revision reconstruction. The Norwegian registry was established in 2004 and reporting has been mandatory since 2017. Overall compliance with the NKLR was 86% in 2017–18. Patients are registered using their unique Norwegian national identification number which links identification of subsequent revision surgery performed within Norway, regardless of the provider. The DKLR was founded in 2005 and similarly records longitudinal outcome of ACL reconstruction within Denmark.

### Participants and predictors

In the index study of NKLR patients [19], four machine learning prediction models were assessed for the ability to predict subsequent revision ACL reconstruction after primary surgery. The four models tested were Cox Lasso, survival random forest, generalized additive model, and gradient boosted regression. These four models are among the most commonly used for this type of analysis. The patients in the NKLR were randomly split into training (75%) and test (25%) sets; the algorithm was developed using the training set of patients, and the performance of the algorithm was assessed with the hold-out test set, previously unseen by the models. The Cox Lasso model was the best-performing of the four tested models and was used for the development of an in-clinic revision-risk calculator (Fig. 1).

Regarding outcome prediction, the four models assessed all the available data in the NKLR to “learn” which factors are associated with—and can be used to predict—which patients will eventually undergo revision surgery. Starting with the 24 total predictor variables in the NKLR, the models eliminated variables which do not significantly improve prediction ability, without sacrificing accuracy. The result was an algorithm developed using the Cox Lasso model that only required five variables (out of the 24) for outcome prediction. The model was well calibrated and demonstrated moderate discriminative ability in predicting revision surgery after primary ACL reconstruction [19].

This study sought to validate the previously developed Cox Lasso model from the NKLR. The Cox Lasso model was selected for validation since it was the best performing model and because some of the variables required for the random forest and gradient boosted regression models were not available in the DKLR. Thus, while the full set of patient characteristics are shown in Table 1, only the five predictors selected by the NKLR Cox Lasso model were used in this validation analysis. The five variables required for outcome prediction using the Cox Lasso model were: patient age at primary surgery, KOOS QoL score at primary surgery, graft choice, femur fixation method, and years between injury and ACL reconstruction.

**Table 1:** Characteristics of Danish registry patients

Variable^a^	*N* = 34,678
Years: surgery to data current date (2021-06-14)	8.3 (4.3)
Missing	1
Revision	1791 (5.2%)
Missing	1
Follow-up time or time to revision	7.6 (4.4)
Missing	1
Age at surgery	29 (10)
Missing	1
Age at injury	27 (10)
Missing	499
Sex	
Female	13,958 (40%)
Male	20,719 (60%)
Missing	1
Pre-surgery KOOS QOL score (out of 10)	3.90 (1.61)
Missing	23,522
Pre-surgery KOOS Sports score (out of 10)	3.80 (2.55)
Missing	23,523
Below median on all pre-surgery KOOS	1868 (17%)
Missing	23,520
Meniscus injury	15,501 (45%)
Cartilage injury	5345 (15%)
Graft choice	
BPTB	3,218 (9.3%)
Hamstring	28,291 (82%)
Unknown/Other	3045 (8.8%)
Missing	124
Tibia fixation device	
Interference screw	30,817 (89%)
Suspension/cortical device	983 (2.8%)
Unknown/Other	2878 (8.3%)
Femur fixation device	
Interference screw	6,072 (18%)
Suspension/cortical device	24,949 (72%)
Unknown/Other	3657 (11%)
Fixation device combination	
Interference screw × 2	5951 (17%)
Interference/Suspension	10 (< 0.1%)
Suspension/cortical device × 2	968 (2.8%)
Suspension/Interference	22,308 (64%)
Unknown/Other	5441 (16%)
Injured side	
Right	17,781 (51%)
Left	16,895 (49%)
Missing	2
Previous surgery on opposite knee	2745 (7.9%)
Missing	108
Previous surgery on same knee	28,809 (83%)
Time injury to surgery (years)	1.65 (3.21)
Missing	712
Systemic antibiotic prophylaxis	34,678 (100%)

^a^Statistics presented: Mean (SD); *n* (%)

For model validation, patients in the DKLR with primary surgery dates from July 2005 through December 2020 were included (*N* = 34,678). To match variables used in the NKLR model, graft choice and femur fixation device were re-coded as shown in Table 1. New variables were defined for time between injury and primary surgery. The Knee Injury and Osteoarthritis Outcome Score (KOOS) Quality of Life (QoL) predictor was scaled to a score out of ten. Patients in the DKLR with missing data for any of the five predictors were excluded from model validation.

### Outcome measures and model performance

The primary outcome in the NKLR Cox Lasso model was probability of revision ACL reconstruction within 1, 2, and/or 5 years. Using R (version: 3.6.1, R Core Team 2019, Vienna, Austria) the NKLR Cox Lasso model was applied to calculate predicted time-to-revision probabilities for all DKLR patients. Performance evaluation included censoring of the time-to-event outcome. “Censoring” refers to the fact that, at any given follow-up time, complete information on outcome is not known for all patients. Some patients have not been in the registry for the requisite number of years, while others have not yet experienced revision and it is unknown when or if they ultimately will.

Performance of the model was assessed using the same metrics as the NKLR study: calibration and concordance at each follow-up time. Calibration refers to the accuracy of the risk estimates and was calculated using a version of the Hosmer–Lemeshow statistic appropriate for censored data [30]. This statistic sums average misclassification in each predicted risk quantile and converts the result into a chi-squared statistic. A larger calibration statistic indicates worse calibration, and statistical significance means the null hypothesis of perfect calibration is rejected. Concordance was computed using Harrell’s C-index [12] at 1, 2, and 5-year follow-up times. The C-index is a generalization of area under the curve (AUC) for censored data that measures the proportion of ranked pairs of observations in which the predicted ranking corresponds with true outcomes. As with AUC, the C-index ranges from 0 to 1 with 1 indicating perfect concordance.

## Results

### Participants

Table 1 describes characteristics of the DKLR population at the time of primary surgery. Patients had an average age at primary surgery of 29 years (SD ± 10) and 60% were male. Hamstring graft was used in 82% of primary surgeries. Of the DKLR patients, 10,922 had complete data for all five variables required by the NKLR Cox Lasso model. Table 2 compares DKLR patients with complete data for these five variables to the NKLR training-data patients with complete data. The large sample sizes produced p-values below the significance threshold on all characteristics, including a few clinically meaningful differences. The DKLR patients were more likely to have hamstring tendon autograft (DKLR: 81%; NKLR: 59%) and suspension/cortical femur fixation (DKLR: 72%; NKLR: 53%). Additionally, the rate of concomitant meniscus (DKLR: 42%; NKLR: 53%) and chondral (DKLR: 14%; NKLR: 23%) injuries were higher in the NKLR cohort, while overall revision rate was higher in the Danish registry patients (DKLR: 6.9%; NKLR: 5.2%). The DKLR patients with complete data on the five required variables were in general similar to those without complete data, particularly on the five required variables (Supplementary Table 1).

**Table 2:** Characteristics of patients with complete data on Norwegian Cox lasso variables

Variable*	Danish *N* = 10,922	Norwegian *N* = 14,161	*P *value**
Years: surgery to data current date (Danish: 06–14-2021; Norwegian: 01–12-2020)	9.3 (4.1)	8.4 (4.1)	< 0.001
Revision	755 (6.9%)	743 (5.2%)	< 0.001
Follow-up time or time to revision	8.4 (4.3)	7.0 (4.2)	< 0.001
Age at surgery	29 (11)	28 (10)	< 0.001
Age at injury	27 (10)	26 (10)	< 0.001
Missing	9	0	
Sex			n.s
Female	4916 (45%)	6376 (45%)	
Male	6006 (55%)	7785 (55%)	
Pre-surgery KOOS QOL score (out of 10)	3.90 (1.61)	3.48 (1.87)	< 0.001
Pre-surgery KOOS Sports score (out of 10)	3.80 (2.55)	4.27 (2.73)	< 0.001
Missing	1	137	
Below median on all pre-surgery KOOS	1825 (17%)	2799 (20%)	< 0.001
Meniscus injury	4584 (42%)	7537 (53%)	< 0.001
Cartilage injury	1579 (14%)	3318 (23%)	< 0.001
Graft choice			< 0.001
BPTB	1133 (10%)	5522 (39%)	
Hamstring	8866 (81%)	8369 (59%)	
Unknown/Other	923 (8.5%)	270 (1.9%)	
Tibia fixation device			< 0.001
Interference screw	9925 (91%)	10,841 (77%)	
Suspension/cortical device	155 (1.4%)	1468 (10%)	
Unknown/Other	842 (7.7%)	1852 (13%)	
Femur fixation device			< 0.001
Interference screw	2025 (19%)	4763 (34%)	
Suspension/cortical device	7891 (72%)	7522 (53%)	
Unknown/Other	1006 (9.2%)	1876 (13%)	
Fixation device combination			< 0.001
Interference screw × 2	1978 (18%)	4645 (33%)	
Interference/Suspension	2 (< 0.1%)	90 (0.6%)	
Suspension/cortical device × 2	153 (1.4%)	1095 (7.7%)	
Suspension/Interference	7218 (66%)	5529 (39%)	
Unknown/Other	1571 (14%)	2802 (20%)	
Injured side			n.s
Right	5512 (50%)	7149 (50%)	
Left	5409 (50%)	7012 (50%)	
Missing	1	0	
Previous surgery on opposite knee	549 (5.0%)	1001 (7.1%)	< 0.001
Missing	27	0	
Previous surgery on same knee	9014 (83%)	2412 (17%)	< 0.001
Time injury to surgery (years)	1.75 (3.34)	1.66 (3.35)	0.040
Systemic antibiotic prophylaxis			< 0.001
Yes	10,922 (100%)	14,089 (99%)	
No	0 (0%)	46 (0.3%)	
Missing	0 (0%)	26 (0.2%)	

^*^Statistics presented: Mean (SD); *n* (%)

^**^Statistical tests: Welch Two Sample *t* test; Pearson’s Chi-squared test

### Model performance

The NKLR Cox Lasso model produced similar concordance with the DKLR population compared to the original NKLR test data (DKLR: 0.68; NKLR: 0.68–0.69). Calibration was poorer for the DKLR population than for the NKLR test data at 1 and 5 years post primary surgery but similar at two years (Table 3).

**Table 3:** Model performance

Probability of Revision	Model	Concordance	Calibration statistic	Calibration p-value
1 year	Original Norwegian Algorithm	0.686	4.89	n.s
	Danish Knee Ligament Registry	0.678	22.24	< 0.001
2 years	Original Norwegian Algorithm	0.684	11.35	0.01
	Danish Knee Ligament Registry	0.676	11.82	0.008
5 years	Original Norwegian Algorithm	0.683	6.19	n.s
	Danish Knee Ligament Registry	0.678	13.98	0.003

## Discussion

The most important finding of this study was that a machine learning algorithm developed from the NKLR demonstrated similar performance when applied to patients from the DKLR. Despite different injury profiles including concomitant meniscus/chondral injury rates and variation in surgical technique trends between the two nations, the concordance was nearly identical to that achieved with the index study of NKLR patients. This suggests that the algorithm is valid for application outside of the initial patient population and represents the first machine learning model for predicting revision ACL reconstruction that has been externally validated. The original model was developed to help guide the clinical discussion regarding surgical options and outcome expectations at a patient-specific level [19].

Machine learning models explore large datasets divided into inputs (predictors) and outputs (outcomes), to establish connections and relationships between them. These relationships may be more complex than could be identified through standard statistical analysis. When a machine learning algorithm can determine a link between the predictors and outcome of interest, it can then create a tool capable of predicting this outcome for other patients. After a prediction model has been developed, the TRIPOD Statement strongly recommends external validation, achieved through evaluation of model performance on new and different groups of patients than were used in the development of the algorithm [6]. However, this important step is uncommonly performed, inhibiting the clinical translation of newly developed models [28].

The original machine learning model was created based on a database including nearly 25,000 patients with 24 variables considered. Four machine learning models were evaluated, and the Cox Lasso model was selected for the development of an in-clinic prediction tool. This tool required the input of only five variables for the prediction of subsequent revision ACL reconstruction risk. Although the performance of this model was assessed using hold-out data that was not included in the learning phase, it only included patients from one nation, limiting its applicability to patients from other countries [19].

This study found that accuracy of the NKLR Cox Lasso model holds when applied to a large data set from another country with different injury characteristics and surgical technique trends. The prediction model demonstrated similar model performance when tested on patients from Denmark that had not been previously seen by the algorithm. It was initially developed using 75% of the patients in the NKLR and validated using the remaining 25%. This study validates the algorithm using an additional 11,000 patients from the DKLR and represents a necessary step toward clinical utility. While this is encouraging, it should be noted that the performance of the model on patients undergoing ACL reconstruction outside of Scandinavia remains unknown. Additionally, there are currently no other published prediction models with which to compare the performance of this model.

Study population variance between the DKLR and NKLR populations may help explain differences in model calibration at one and five years post primary surgery. The DKLR patients with complete data had higher proportions of hamstring tendon autograft and suspension/cortical femur fixation than patients in the NKLR test data. Both these variables are used in the NKLR Cox Lasso model. Thus, the relationship between graft choice and/or femur fixation and revision risk codified in the model may not be as accurate for patient populations with a substantially different distribution on these variables, such as those in the DKLR. Regarding the fact that the validation data set was limited to approximately one-third of the overall DKLR registry population due to missing values for the required predictors, the objective of this paper was to test the machine learning model on a new population and the inclusion of nearly 11,000 patients represents a suitable data set for this purpose.

While this novel technique represents a new frontier for health-related research, limitations regarding the clinical utility of machine learning algorithms remain. Most importantly, the quality of the model is largely related to the quality of the data that it is developed from. The concordance of the revision ACL prediction tool is moderate based on both the initial and subsequent validation studies. As noted in the original paper, this may be related to data quality since several risk factors for failure of ACL reconstruction are not captured in the NKLR [19]. Examples of these factors include radiographic variables such as tibial slope and coronal alignment [2–4, 10, 15, 20, 31], physical examination and rehabilitation details [11, 14, 18, 22], and surgical technique factors such as tunnel position [16] and graft size [1, 7, 17]. The addition of these variables into the national knee ligament registers may improve future machine learning prediction endeavours.

There is an additional limitation concerning this external validation study. Since pre-operative KOOS QoL score at the time of surgery was one of the input variables required for outcome prediction, all patients in the DKLR without a pre-operative KOOS score were excluded from the analysis. This resulted in the exclusion of approximately two-thirds of the patients contained in the DKLR since pre-surgical compliance with patient reported outcome measures is relatively low in the registry. Despite this, nearly 11,000 patients were still included in the model evaluation which is sufficient for validation.

Machine learning analysis of large health-care registries have the potential for great impact on patient care. These advanced statistical techniques can assess and interpret large volumes of data and recognize complex associations between predictor variables and patient-specific outcome. The resulting algorithm, as is the case with the present study, can be implemented into clinical care as an adjunct for the orthopaedic surgeon. Supplementing their personal experience and interpretation of the relevant risk factors, clinicians can use this in-clinic calculator to individualize their discussions and quantify the risk of revision ACL reconstruction for their patients.

## Conclusion

The NKLR machine learning algorithm demonstrated similar performance when applied to patients from the DKLR, suggesting that it is valid for application outside of the initial patient population. This represents the first machine learning model for predicting revision ACL reconstruction that has been externally validated. Clinicians can use this in-clinic calculator to estimate revision risk at a patient specific level when discussing outcome expectations pre-operatively. While encouraging, it should be noted that the performance of the model on patients undergoing ACL reconstruction outside of Scandinavia remains unknown.

## Supplementary Information

Below is the link to the electronic supplementary material.

Supplementary file1 (PDF 68 KB)

Supplementary file2 (DOCX 21 KB)
