# Supplementary material for: Machine learning algorithm to predict anterior cruciate ligament revision demonstrates external validity
Source: Knee Surg Sports Traumatol Arthrosc. 2022 Jan 1;30(2):368–75. doi: 10.1007/s00167-021-06828-w (PMC8866372; doi:10.1007/s00167-021-06828-w)
Supplement: Supplementary file 1 — Supplementary file1 (PDF 68 KB) [file 167_2021_6828_MOESM1_ESM.pdf]

TRIPOD Checklist: Prediction Model Validation

Section/Topic	Item	Checklist Item	Page
**Title and abstract**			
Title	1	Identify the study as developing and/or validating a multivariable prediction model, the target population, and the outcome to be predicted.	1
Abstract	2	Provide a summary of objectives, study design, setting, participants, sample size, predictors, outcome, statistical analysis, results, and conclusions.	2-3
**Introduction**			
Background and objectives	3a	Explain the medical context (including whether diagnostic or prognostic) and rationale for developing or validating the multivariable prediction model, including references to existing models.	4
	3b	Specify the objectives, including whether the study describes the development or validation of the model or both.	5
**Methods**			
Source of data	4a	Describe the study design or source of data (e.g., randomized trial, cohort, or registry data), separately for the development and validation data sets, if applicable.	5-7
	4b	Specify the key study dates, including start of accrual; end of accrual; and, if applicable, end of follow-up.	5-7
Participants	5a	Specify key elements of the study setting (e.g., primary care, secondary care, general population) including number and location of centres.	5-7
	5b	Describe eligibility criteria for participants.	5-7
	5c	Give details of treatments received, if relevant.	5-7
Outcome	6a	Clearly define the outcome that is predicted by the prediction model, including how and when assessed.	7-8
	6b	Report any actions to blind assessment of the outcome to be predicted.	N/A
Predictors	7a	Clearly define all predictors used in developing or validating the multivariable prediction model, including how and when they were measured.	7
	7b	Report any actions to blind assessment of predictors for the outcome and other predictors.	N/A
Sample size	8	Explain how the study size was arrived at.	7
Missing data	9	Describe how missing data were handled (e.g., complete-case analysis, single imputation, multiple imputation) with details of any imputation method.	7
Statistical analysis methods	10c	For validation, describe how the predictions were calculated.	7-8
	10d	Specify all measures used to assess model performance and, if relevant, to compare multiple models.	7-8
	10e	Describe any model updating (e.g., recalibration) arising from the validation, if done.	N/A
Risk groups	11	Provide details on how risk groups were created, if done.	N/A
Development vs. validation	12	For validation, identify any differences from the development data in setting, eligibility criteria, outcome, and predictors.	8
**Results**			
Participants	13a	Describe the flow of participants through the study, including the number of participants with and without the outcome and, if applicable, a summary of the follow-up time. A diagram may be helpful.	8-9
	13b	Describe the characteristics of the participants (basic demographics, clinical features, available predictors), including the number of participants with missing data for predictors and outcome.	9
	13c	For validation, show a comparison with the development data of the distribution of important variables (demographics, predictors and outcome).	8-9
Model performance	16	Report performance measures (with CIs) for the prediction model.	Table 3
Model-updating	17	If done, report the results from any model updating (i.e., model specification, model performance).	N/A
**Discussion**			
Limitations	18	Discuss any limitations of the study (such as nonrepresentative sample, few events per predictor, missing data).	11-12
Interpretation	19a	For validation, discuss the results with reference to performance in the development data, and any other validation data.	10-11
	19b	Give an overall interpretation of the results, considering objectives, limitations, results from similar studies, and other relevant evidence.	10-12
Implications	20	Discuss the potential clinical use of the model and implications for future research.	11-12
**Other information**			
Supplementary information	21	Provide information about the availability of supplementary resources, such as study protocol, Web calculator, and data sets.	4
Funding	22	Give the source of funding and the role of the funders for the present study.	8

We recommend using the TRIPOD Checklist in conjunction with the TRIPOD Explanation and Elaboration document.
