# Supplementary material for: Machine learning algorithm to predict anterior cruciate ligament revision demonstrates external validity
Source: Knee Surg Sports Traumatol Arthrosc. 2022 Jan 1;30(2):368–75. doi: 10.1007/s00167-021-06828-w (PMC8866372; doi:10.1007/s00167-021-06828-w)
Supplement: Supplementary file 2 — Supplementary file2 (DOCX 21 KB) [file 167_2021_6828_MOESM2_ESM.docx]

**Supplementary Table 1: Comparison of Danish registry patients with complete vs. incomplete data on Norwegian Cox lasso variables**

(Yellow highlights show Norwegian cox lasso variables)

| **Variable*** | **Complete**  **N = 10,922** | **Incomplete**  **N = 23,756** | **P-value**** |
| --- | --- | --- | --- |
| Years: surgery to data current date (06-14-2021) | 9.3 (4.1) | 7.9 (4.3) | <0.001 |
| Missing | 0 | 1 |  |
| Revision | 755 (6.9%) | 1,036 (4.4%) | <0.001 |
| Missing | 0 | 1 |  |
| Follow-up time or time to revision | 8.4 (4.3) | 7.2 (4.3) | <0.001 |
| Missing | 0 | 1 |  |
| Age at surgery | 29 (11) | 28 (10) | 0.006 |
| Missing | 0 | 1 |  |
| Age at injury | 27 (10) | 27 (10) | n.s. |
| Missing | 9 | 490 |  |
| Sex |  |  | <0.001 |
| Female | 4,916 (45%) | 9,042 (38%) |  |
| Male | 6,006 (55%) | 14,713 (62%) |  |
| Missing | 0 | 1 |  |
| Pre-surgery KOOS QOL score (out of 10) | 3.90 (1.61) | 3.74 (1.58) | n.s. |
| Missing | 0 | 23,522 |  |
| Pre-surgery KOOS Sports score (out of 10) | 3.80 (2.55) | 3.76 (2.57) | n.s. |
| Missing | 1 | 23,522 |  |
| Below median on all pre-surgery KOOS | 1,825 (17%) | 43 (18%) | n.s. |
| Missing | 0 | 23,520 |  |
| Meniscus injury | 4,584 (42%) | 10,917 (46%) | <0.001 |
| Cartilage injury | 1,579 (14%) | 3,766 (16%) | <0.001 |
| Graft choice |  |  | <0.001 |
| BPTB | 1,133 (10%) | 2,085 (8.8%) |  |
| Hamstring | 8,866 (81%) | 19,425 (82%) |  |
| Unknown/Other | 923 (8.5%) | 2,122 (9.0%) |  |
| Missing | 0 | 124 |  |
| Tibia fixation device |  |  | <0.001 |
| Interference screw | 9,925 (91%) | 20,892 (88%) |  |
| Suspension/cortical device | 155 (1.4%) | 828 (3.5%) |  |
| Unknown/Other | 842 (7.7%) | 2,036 (8.6%) |  |
| Femur fixation device |  |  | <0.001 |
| Interference screw | 2,025 (19%) | 4,047 (17%) |  |
| Suspension/cortical device | 7,891 (72%) | 17,058 (72%) |  |
| Unknown/Other | 1,006 (9.2%) | 2,651 (11%) |  |
| Fixation device combination |  |  | <0.001 |
| Interference screw x2 | 1,978 (18%) | 3,973 (17%) |  |
| Interference/Suspension | 2 (<0.1%) | 8 (<0.1%) |  |
| Suspension/cortical device x2 | 153 (1.4%) | 815 (3.4%) |  |
| Suspension/Interference | 7,218 (66%) | 15,090 (64%) |  |
| Unknown/Other | 1,571 (14%) | 3,870 (16%) |  |
| Injured side |  |  | 0.043 |
| Right | 5,512 (50%) | 12,269 (52%) |  |
| Left | 5,409 (50%) | 11,486 (48%) |  |
| Missing | 1 | 1 |  |
| Previous surgery on opposite knee | 549 (5.0%) | 2,196 (9.3%) | <0.001 |
| Missing | 27 | 81 |  |
| Previous surgery on same knee | 9,014 (83%) | 19,795 (83%) | n.s. |
| Time injury to surgery (years) | 1.75 (3.34) | 1.60 (3.14) | <0.001 |
| Missing | 0 | 712 |  |
| Systemic Antibiotic Prophylaxis | 10,922 (100%) | 23,756 (100%) |  |

*Statistics presented: Mean (SD); n (%)

**Statistical tests: Welch Two Sample t-test; Pearson's Chi-squared test; Fisher’s exact test for fixation device combination variable
